# Predicting immunotherapy outcomes in patients with MSI tumors using NLR and CT global tumor volume

**DOI:** 10.3389/fonc.2022.982790

**Published:** 2022-10-25

**Authors:** Younes Belkouchi, Laetitia Nebot-Bral, Littisha Lawrance, Michele Kind, Clémence David, Samy Ammari, Paul-Henry Cournède, Hugues Talbot, Perrine Vuagnat, Cristina Smolenschi, Patricia L. Kannouche, Nathalie Chaput, Nathalie Lassau, Antoine Hollebecque

**Affiliations:** ^1^ Laboratoire d’Imagerie Biomédicale Multimodale Paris-Saclay (BIOMAPS), UMR 1281, Université Paris-Saclay, Inserm, CNRS, CEA, Villejuif, France; ^2^ OPtimisation Imagerie et Santé (OPIS), Inria, CentraleSupélec, Université Paris-Saclay, Gif-Sur-Yvette, France; ^3^ UMR9019 - CNRS, Intégrité du Génome et Cancer, Université Paris-Saclay, Gustave Roussy, Villejuif, France; ^4^ Département d’Imagerie Médicale, Institut Bergonié, Bordeaux, France; ^5^ Département d’Imagerie, Gustave Roussy, Université Paris Saclay, Villejuif, France; ^6^ Mathématiques et Informatique pour la Complexité et les Systèmes (MICS), CentraleSupélec, Université Paris-Saclay, Gif-Sur-Yvette, France; ^7^ Département d’Innovation Thérapeutique et d’Essais Précoces (DITEP), Gustave Roussy, Université Paris Saclay, Villejuif, France; ^8^ Université Paris-Saclay, Faculté de Pharmacie, Chatenay-Malabry, France; ^9^ Laboratoire d’Immunomonitoring en Oncologie, Gustave Roussy, Villejuif, France

**Keywords:** mismatch repair (MMR) deficiency, immunotherapy, CT scan, tumor volume, biomarker, anti-PD1, neutrophile-to-lymphocyte ratio (NLR), anti PDL1

## Abstract

**Background:**

Anti-PD-(L)1 treatment is indicated for patients with mismatch repair-deficient (MMRD) tumors, regardless of tumor origin. However, the response rate is highly heterogeneous across MMRD tumors. The objective of the study is to find a score that predicts anti-PD-(L)1 response in patients with MMRD tumors.

**Methods:**

Sixty-one patients with various origin of MMRD tumors and treated with anti-PD-(L)1 were retrospectively included in this study. An expert radiologist annotated all tumors present at the baseline and first evaluation CT-scans for all the patients by circumscribing them on their largest axial axis (single slice), allowing us to compute an approximation of their tumor volume. In total, 2120 lesions were annotated, which led to the computation of the total tumor volume for each patient. The RECIST sum of target lesions’ diameters and neutrophile-to-lymphocyte (NLR) were also reported at both examinations. These parameters were determined at baseline and first evaluation and the variation between the first evaluation and baseline was calculated, to determine a comprehensive score for overall survival (OS) and progression-free survival (PFS).

**Results:**

Total tumor volume at baseline was found to be significantly correlated to the OS (p-value: 0.005) and to the PFS (p-value:<0.001). The variation of the RECIST sum of target lesions’ diameters, total tumor volume and NLR were found to be significantly associated to the OS (p-values:<0.001, 0.006,<0.001 respectively) and to the PFS (<0.001,<0.001, 0.007 respectively). The concordance score combining total tumor volume and NLR variation was better at stratifying patients compared to the tumor volume or NLR taken individually according to the OS (pairwise log-rank test p-values: 0.033,<0.001, 0.002) and PFS (pairwise log-rank test p-values: 0.041,<0.001, 0.003).

**Conclusion:**

Total tumor volume appears to be a prognostic biomarker of anti-PD-(L)1 response to immunotherapy in metastatic patients with MMRD tumors. Combining tumor volume and NLR with a simple concordance score stratifies patients well according to their survival and offers a good predictive measure of response to immunotherapy.

## Introduction

Immunotherapy, and particularly Immune checkpoint blockades (ICBs) harness a patient’s own immune response against a tumor by blocking inhibitory signaling molecules expressed on T-cells, thereby strengthening their antitumor response. ICBs such as anti-PD-1 monoclonal antibodies, have revolutionized the management of several cancers (1–3). Over the last few decades, the number of articles regarding immunotherapy has doubled every two years (4) and the clinical trials intend to apply this new anticancer weapon in the treatment of the highest number of patients possible. Several clinical trials have shown the efficacy of anti-PD1 and PD-L1 (anti-PD-(L)1) antibodies on tumors with microsatellite instability (MSI) phenotype in patients (5–7). Considering all types of mutations, MSI tumors display a high mutation rate, with 12-40 mutations/Mb (8, 9). This high mutational burden is supposed to make tumors immunogenic, and this rational supports the first approval for anti-PD-1 antibodies for patients with metastatic MSI cancers, regardless of the tumor type. MSI tumors represent 15% and 30% of colorectal and endometrial cancers, respectively (10). Thus, most of the clinical studies that have evaluated ICB efficacy recruited patients with MSI colorectal and endometrial cancer (11–13). Nonetheless, MSI tumors have been identified in more than 15 different tumors types and, Azad et al., and Marabelle et al., have reported a highly variable response to anti-PD-(L)1 across MSI tumors, ranging from 0% in MSI brain tumors to 57.1% in MSI endometrial cancers (14, 15). The underlying molecular mechanisms to explain this heterogeneity remains elusive. There is an imperative need to find biomarkers that vary before or early-on after ICB initiation. In this regard, we have recently proven that the variation of Neutrophil to Lymphocyte ratio (NLR_change_) at 2 months after treatment initiation can discriminate patients with progressive disease from those who respond to PD(L)-1 inhibition, regardless of the anatomic sites of mismatch repair-deficient (MMRD) (16). More precisely, the increase of NLR was significantly associated with better overall survival and progression-free survival (HR_logrank_=0.3755 (%95CI= 0.2013-0.7004); HR_logrank_=0.5173 (%95CI= 0.2895-0.9241) respectively). Interestingly, the change in NLR was observed earlier (2 months) compared to the tumor changes described by imaging data (CT, MRI), thus allowing NLR_change_ to be used for an earlier evaluation of response to treatment.

Other parameters have been described in the literature as biomarker candidates (17, 18). They are classified as tumor-based biomarkers, circulating factors and host-related markers. Tumor-based biomarkers gather the tumor mutational burden (TMB), PD-L1 expression and populations of the immune system that can invade the tumor (tumor microenvironment, TME) and exert antitumoral or pro-tumoral function. The balance between the Ying and Yang of the immune system is being widely studied using scores such as Immunophenoscore (19) and immune set point (20) that quantify tumor immune infiltration (cytotoxic T cells, effector T cell, memory T cell, NK cells, macrophages, T regulatory cells) and chemokine profile. Among circulating factors, the circulating tumor DNA, and cytokines such as IL8 are also being extensively studied (21). Finally, the host parameters and particularly the intestinal commensal microbiota could modify ICB response (22). Moreover, other parameters such as those based on medical images could be important factors as well. The tumor burden can be easily assessed relative to other tumor biomarkers through medical imaging and might have important clinical implications as well (23).

Based on both the number and size of metastases as well as the number of organs affected, the Response Evaluation Criteria In Solid Tumors (RECIST 1.1) (24) is considered as the “gold standard” currently to evaluate CT scans at baseline and post-treatment. The assessment of change in tumor size according to RECIST 1.1 is used for the clinical evaluation of objective response, stable disease and disease progression (24). However, at baseline, the role of tumor size in predicting the outcome of ICB response has been poorly investigated and remains a matter of debate (23). Furthermore, RECIST 1.1 evaluation has several limitations. It considers no more than 5 lesions in total, a maximum of 2 lesions per organ. Thus, the accurate quantification of the metastatic dissemination is likely underestimated. Additionally, atypical responses have been observed in the case of immunotherapy, such as pseudo-progression or hyper-progression (25, 26). Pseudo-progression led to the development of the iRECIST protocol, which requires a validation at least one month after progression to confirm if the progression is a real or pseudo progression (27). While the incidence rate for the phenomenon is low, this emphasizes the need for a prognostic biomarker to evaluate the response to immunotherapy. Moreover, Kuhl et al. have shown a discordance between different readers when assessing tumor response, if the chosen target lesions were different (κ = 0.58; 95% CI: 0.54, 0.62) (28). Hence, we decided to extensively characterize the tumor burden characteristics and NLR in patients treated with anti-PD-(L)1. Our study aims at finding a score combining the comprehensive tumor volume and NLR, for an earlier evaluation of response to immunotherapy, in patients with MMRD tumors.

## Materials and methods

### Ethics statement

All patients included in this study received at least one previous line of anti-PD-(L)1 therapy. They were enrolled from November 2014 to October 2020 at Gustave Roussy and provided informed consent before enrollment in these studies. The study is reported in Health Data Hub.

### Patients’ characteristics

This retrospective study enrolled 61 patients with metastatic cancer of various histological types treated with anti-PD-(L)1. The patients included were all screened for MMRD by the IHC staining to determine the presence/absence of the main MMR proteins (*MSH2, MSH6, PMS2, MLH1*). Two CT-scans were required for this cohort: a baseline preceding the immunotherapy and the other at first evaluation. The median time between baseline and first evaluation was 2.1 months (IQR: 1.6 – 2.6 months). Blood tests were also performed at the two evaluations, where the absolute neutrophil count and the absolute lymphocyte count were assessed. The Neutrophil-to-Lymphocyte (NLR) ratio is defined as the ratio between absolute neutrophil count and absolute lymphocyte count.

### Radiological features & tumor burden

**Figure 1:**
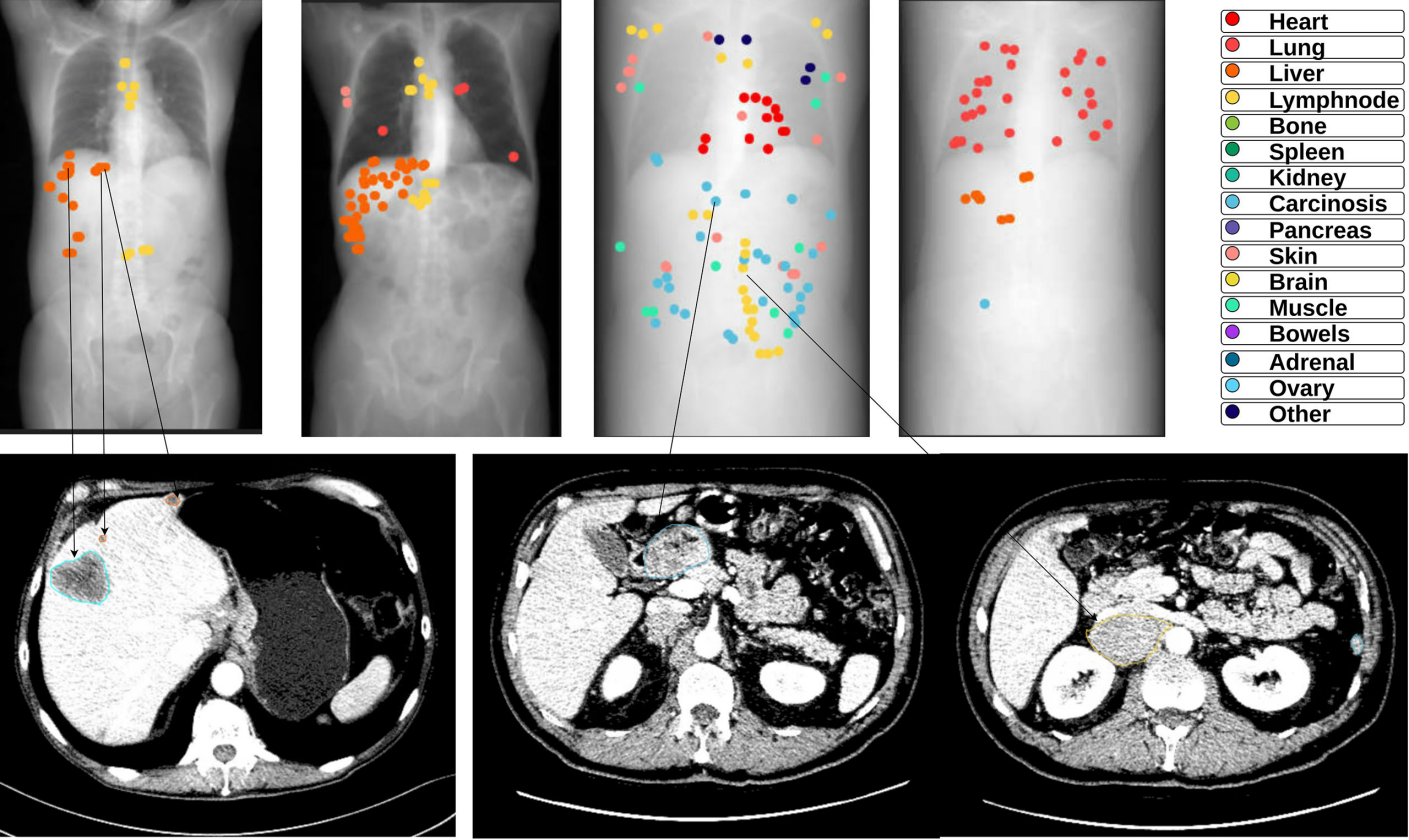
Examples of annotations on CT scans of different metastatic patients at baseline. The upper part contains an overview (CT Scout image) of the thorax-abdomen-pelvis CT scans that were fully annotated. Each colored dot in the overview is an annotation on its corresponding axial slice, the color indicates the tumor location or type (See Figure legend). The lower part shows a few examples of the tumor annotations on the axial slice.

The CT-scans were analyzed by an expert radiologist who annotated all lesions in chest-abdomen-pelvis scans using an internal annotation software called Spyd. The annotations were performed with the following constraints: a) Precise freehand circumscribed annotation of all lesions in the body at their largest axial position (single slice), b) Lymph nodes were only considered a lesion if their smallest axis measured more than 10 mm, c) all pulmonary micronodules measuring more than 3 mm were annotated, d) Each annotation contained a label describing the location of the tumor. The tumor labels used were heart, lung, liver, lymph node, bone, spleen, kidney, carcinosis, ovary, pancreas, skin/soft tissue, adrenal, brain, muscle, bowels and other. The scans at both evaluations were annotated independently for each patient (**Figure 1**). These annotations allowed us to compute the shape features: Major Axis, Minor Axis and Mesh Surface, using Pyradiomics software (29). The surface is computed using the following formulas: $A\;=\;\Sigma \frac{1}{2}Oa_{i}\times Ob_{i}$ where *O*_*i*_ *a*_*i*_ and *O*_*i*_ *b*_*i*_ are edges of the ith triangle in the mesh, formed by vertices *a*_*i*_ , *b*_*i*_ of the perimeter and the origin O. The Major Axis and Minor Axis are computed using the two smallest singular values on the ROI-enclosed ellipsoid: $major\;=\;4\sqrt{\lambda_{major}}$ and $minor\;=\;4\sqrt{\lambda_{minor}}.$ The approximate 3D volume for each metastasis was obtained with the following formula: Tumor volume = 2/3 x Surface x Minor Axis. The total tumor volume for each patient is computed using the sum of the approximate 3D volume of each metastasis.

The RECIST 1.1 evaluation done by another radiologist was also acquired, as well as the diameters of the target lesions using the RECIST 1.1 guidelines, and their sum was computed.

### Dynamic changes

To determine a prognostic biomarker, the parameters were evaluated at baseline. The variation of the parameters between baseline and first evaluation were calculated to determine predictive biomarkers for immunotherapy response. The variation was defined as: variation = $\frac{parameter_{E1}\;-\;parameter_{BL}}{parameter_{BL}}\;\times \;100$ , where E1 and BL stand for Evaluation 1 (first radiological evaluation) and baseline respectively. Only an increase (variation > 0%) or decrease (variation< 0%) of the parameters was evaluated in this study. Five parameters were studied at baseline and in variation: Sum of RECIST 1.1 target lesion diameters (RECIST), total tumor volume (Volume), total number of lesions (Number Lesions), number of organs affected by lesions (Organs involved) and NLR.

### Statistical analysis

The main objective was to evaluate the biomarkers based on Overall survival (OS) and Progression-free survival (PFS). OS was defined as the time between the start of immunotherapy (first injection) and death from any cause. PFS was defined as the time between immunotherapy initiation and tumor progression based on RECIST 1.1 or death, whichever occurred first. The median and interquartile range (IQR) were used to report the distribution of variables. Spearman correlation was used to evaluate the dependency between variables. Variables were considered independent if their coefficients were lower than 0.8. When building our final score, only the independent variables were used. The Kaplan-Meier method was used for univariate survival analysis. The Cox model was used for multivariate data analysis, and the hazard ratio (HR) was computed for each covariate. The log-rank test was used to compare the survival distribution of the categories obtained from the univariate parameters, and to compute the p-value to assess the significance of the comparison. The cut-offs chosen for the binarization of continuous variables was the median approximated to the nearest integer for all parameters but NLR. The cut-off for NLR at baseline was 5 as previously described by others (30, 31). All the statistical analyses were performed using the Python language environment version 3.10, with the lifelines v0.25.7 (32), PyRadiomics v3.0.1 (29) and pandas v1.1.5 (33) software packages. Figures were generated using Prism 9 (GraphPad, San Diego, CA, USA).

## Results

### Patients’ characteristics

This retrospective study includes 61 patients treated with immunotherapy between 2015 and 2020, with MSI/MMRD tumors. Each patient had 2 CT scans that were evaluated. In total, 61,364 slices were analyzed to circumscribe 2120 lesions at their largest axial axis. Patients’ baseline characteristics are listed in **Table 1**. The median age of all patients was 64. The patients had different primitive cancers, the most notable ones being Colorectal Cancer (42%) and Endometrial (21%). Patients were equally treated using either anti-PD-1 or anti-PD-(L)1. There was no significant difference in OS or PFS according to the therapy used (**Supplementary Figure 1**). The overall response rate of patients was 51%. The median time to progression and response were 3.2 (IQR: 1.5-9.4) and 3 (IQR: 2.0-6.5) months respectively. 34 patients were right censored for the OS, and 27 for the PFS.

**Table 1:** Baseline patients’ characteristics.

Characteristics	No.	%
Total patients	61	100
Age		
Median	64	
IQR	52-70	
Sex		
Male	29	48
Female	32	52
Tumor Type		
Colorectal Cancer	26	43
Endometrial	13	21
Gastric	7	11
Small Intestine	5	8
Prostate	2	3
Ovarian	2	3
Esophagus	2	3
Pancreas	2	3
Adrenal Gland	1	2
Uterine cervix	1	2
Treatment		
Anti-PD-1	26	43
Anti-PD-L1	35	57
Responder		
Yes	31	51
No	30	49
Time to progression (months) Median 95% CI median Time to response (months) Median 95% CI median	3.2 1.8-6.1 3.0 2.1-6.0	

A patient is considered as a responder if the best response during treatment was either Partial or Complete. Stable disease patients were considered non-responders.

### Baseline parameters

**Figure 2:**
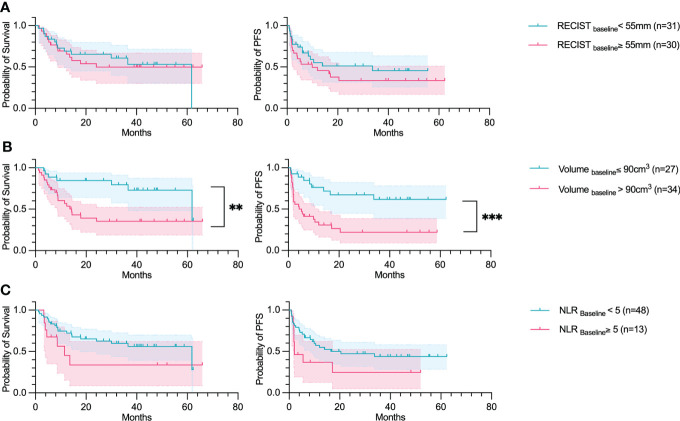
Volume_baseline_ predicts OS and PFS. Overall survival and Progression-free survival plotted using a Kaplan Meier estimation with their respective confidence intervals (95%). The parameters studied are: **(A)** The sum of RECIST diameters (RECIST); **(B)** Total tumor volume (Volume); and **(C)** Neutrophil-to-lymphocyte ratio (NLR). For Kaplan–Meier estimation, tick marks represent data censored at the time of the last imaging assessment and statistical analyses were performed using Log-rank (Mantel-Cox) test. Symbol significance: * p ≤ 0.05; ** p ≤ 0.01; *** ≤0.001, **** ≤0.0001.

Five parameters extracted from the baseline CT-scan, namely the comprehensive tumor volume (Volume_baseline_), the number of organs affected at baseline (Organ count_baseline_), the NLR (NLR_baseline_), the total number of lesions (Number lesions_baseline_) and the RECIST diameters’ sum (RECIST_baseline_), were analyzed and correlated with the OS and PFS in a univariate study. The median cut-offs were: for Volume_baseline_ 90cm3 (~5.6 cm in diameter); 3 organs for Organ count_baseline_; Number lesions_baseline_ was 10 lesions; and 5.5 cm for RECIST_baseline_. The most significant variable was the Volume_baseline_ (OS p-value: 0.005, PFS p-value:<0.001) (**Figure 2**). The RECIST_baseline_ and NLR_baseline_ were not significant at this point of time. However, the variable significant at baseline, correlating to the OS and PFS was Number lesions_baseline_(OS p-value: 0.032, PFS p-value: 0.009) (**Supplementary Figure 2**). The Organ count_baseline_ significantly correlated only to the OS (p-value: 0.005).

**Figure 3:**
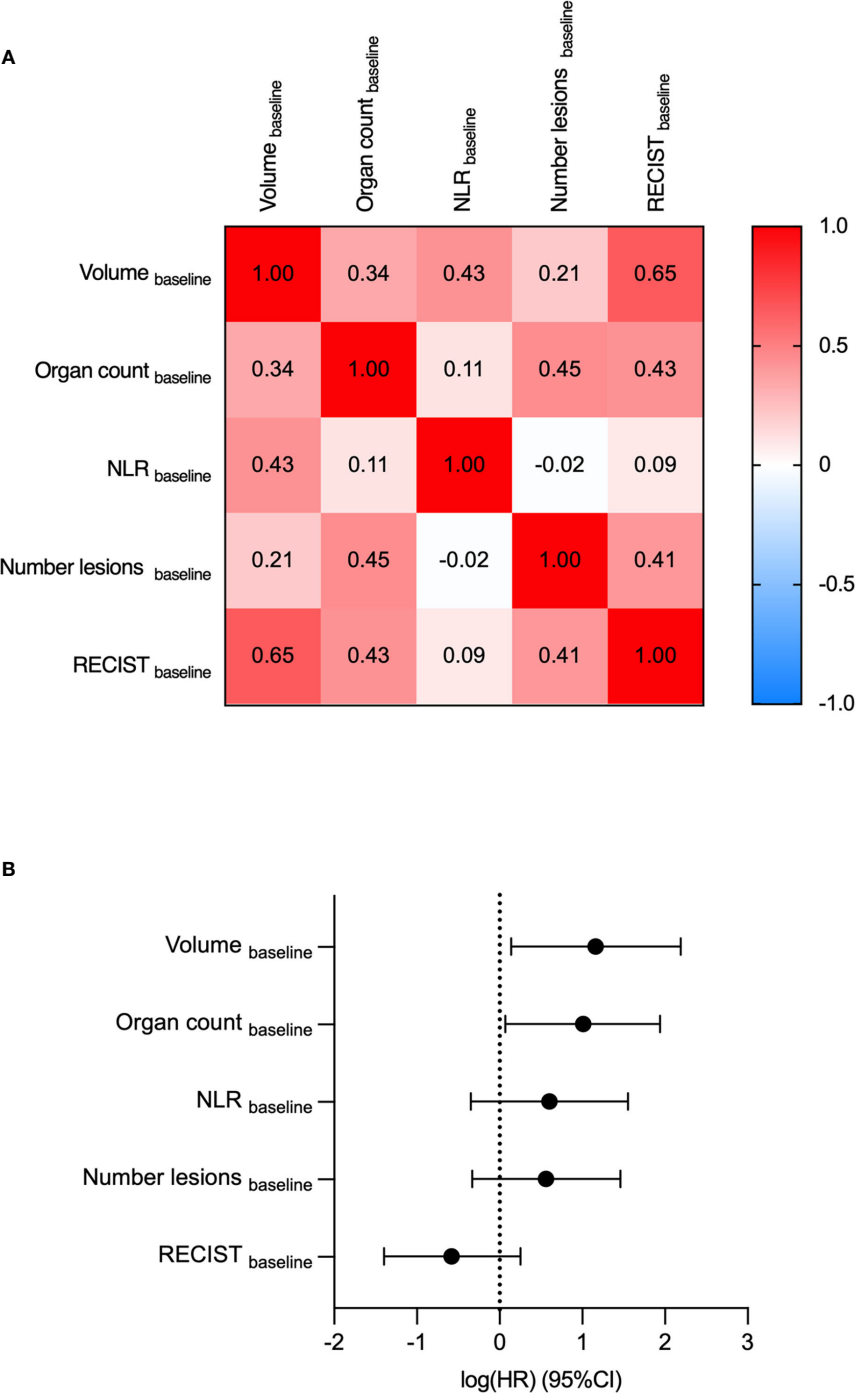
Multivariate analyses determine Volume_baseline_ and Organ count_baseline_ as the most predictive variables for OS. Volume_baseline_, Organ count_baseline_, NLR_baseline_, Number of lesions_baseline_ and RECIST_baseline_ were compared by multivariate analyses. **(A)** The Spearman correlation coefficients of the five parameters. **(B)** Log-hazard ratio (HR) according to each covariate. Data are shown as HR (95% CI).

A Cox regression hazard model was fitted on the 5 parameters to analyze them in a multivariate setting. Two parameters were found to be significant for predicting the OS: Volume_baseline_ (HR=3.2, p-value=0.03) and the Organ count_baseline_, (HR=2.7, p-value=0.03) (**Figure 3**). The features were found to be independent of each other according to the Spearman correlation test. Comparing the correlation among the variables, Volume_baseline_ and RECIST_baseline_ had the highest correlation coefficient (0.65).

### Variation parameters

**Figure 4:**
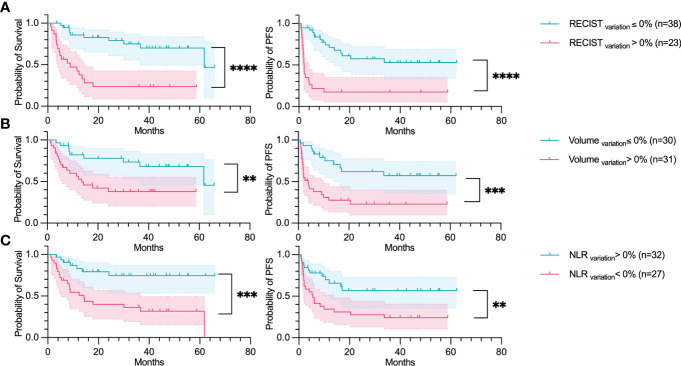
RECIST_variation_, Volume_variation_ and NLR_variation_ predicts OS and PFS. Overall survival and Progression-free survival plotted using a Kaplan-Meier estimation with their respective confidence intervals (95%). The parameters studied are: **(A)** The increase in the sum of RECIST diameters (RECIST); **(B)** Increase of total tumor volume (Volume); and **(C)** Increase of neutrophil-to-lymphocyte ratio (NLR). For Kaplan–Meier estimation, tick marks represent data censored at the time of the last imaging assessment and statistical analyses were performed using Log-rank (Mantel-Cox) test. Symbol significance: * p ≤ 0.05; ** p ≤ 0.01; *** ≤0.001, **** ≤0.0001.

The five parameters as at the baseline were also analyzed at the first evaluation. Their increase or decrease were then calculated in search of predictive biomarkers of response. The RECIST sum of diameters variation (RECIST_variation_) was highly correlated to both OS and PFS (OS p-value:<0.001, PFS p-value:<0.001) (**Figure 4**). The variation of the comprehensive tumor volume (Volume_variation_), the NLR variation (NLR_variation_) were also found to be significantly correlated to the OS (p-values: 0.006, and<0.001 respectively) and PFS (p-value:<0.001, and 0.007 respectively).

**Figure 5:**
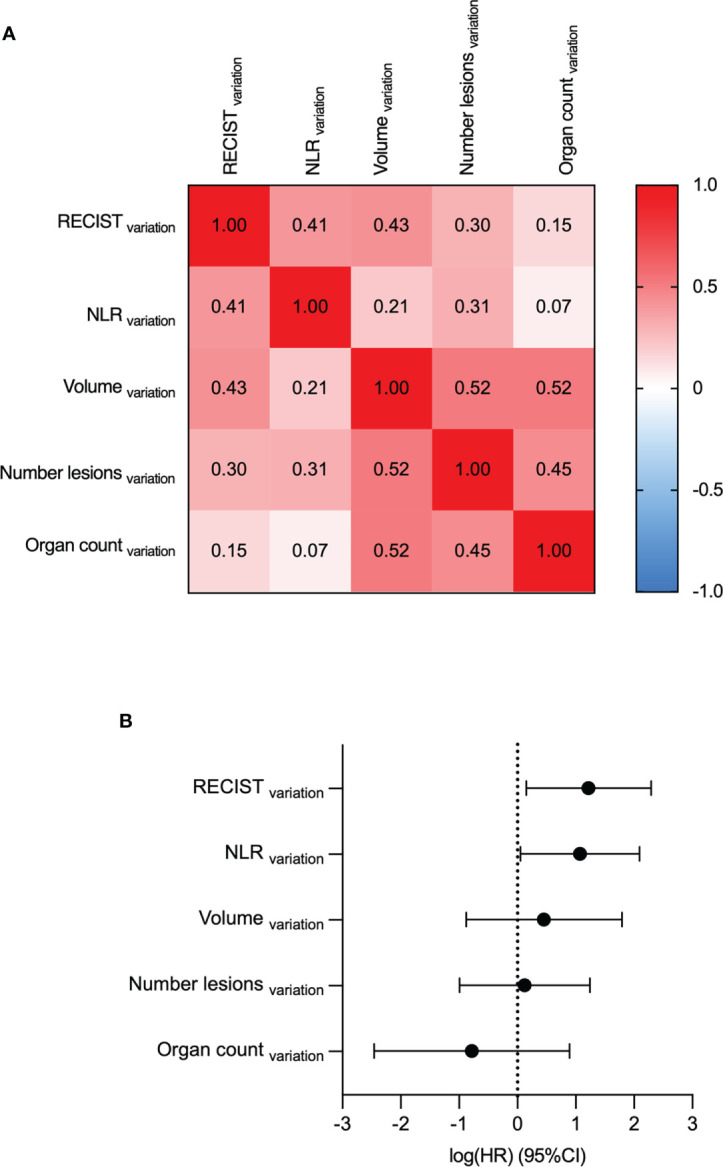
Multivariate analyses determine RECIST_variation_ and NLR_variation_ as the most predictive variables for OS. RECIST_variation_, NLR_variation_, Volume_variation_ Number lesions_variation_ and Organ count_variation_ were compared by multivariate analyses. **(A)** The Spearman correlation coefficients. **(B)** Log-hazard ratio (HR) according to each covariate. Data are shown as HR (95% CI).

A Cox regression hazard model was fitted on the variation of the parameters to analyze them in a multivariate setting. Two parameters were found to be significant: The RECIST_variation_ (HR=3.4, p-value=0.02) and NLR_variation_ (HR=2.9, p-value=0.04) (**Figure 5**). None of the features were found to be correlated according to the Spearman correlation test. The features with the highest correlation coefficient were the tumor volume (Volume_variation_) and the increase in the number of organs affected (Organ count_variation_), with a coefficient of 0.52.

### Concordance score

**Figure 6:**
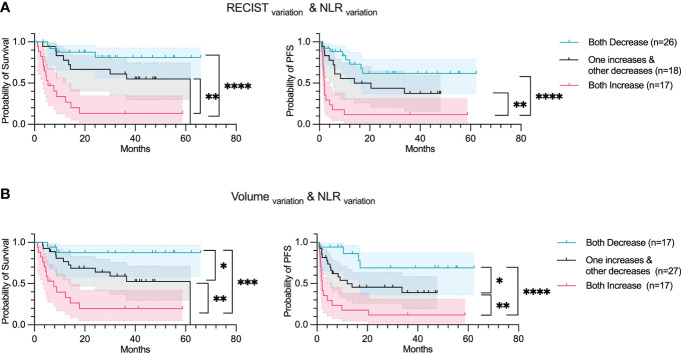
Concordance score predicts OS and PFS. Overall survival and Progression-free survival plotted using a Kaplan-Meier estimation with their respective confidence intervals (95%). The parameters studied are: **(A)** Concordance score using RECIST_variation_ & NLR_variation_. **(B)** Concordance score using Tumor volume_variation_ & NLR_variation_. For Kaplan–Meier estimation, tick marks represent data censored at the time of the last imaging assessment and statistical analyses were performed using Log-rank (Mantel-Cox) test. Symbol significance: * p ≤ 0.05; ** p ≤ 0.01; *** ≤0.001, **** ≤0.0001.

A concordance score was computed for NLR_variation_ and RECIST_variation_; as well as for NLR_variation_ and Volume_variation_. A concordance score reflects a mutual increase, a mutual decrease, and a discordance in the two parameters. Both concordance scores were found to be significant, with the Volume_variation_ and NLR_variation_ found to be slightly more significant than RECIST_variation_ and NLR_variation_ (p< 0.05 log-rank test between mutual decrease and discordance, corresponding to both OS and PFS) (**Figure 6**).

## Discussion

Understanding factors that contribute to ICB effectiveness is crucial to a broader application of these therapies. By investigating the biological and clinical parameters of the study and gathering patients diagnosed with different histological MSI tumors treated with PD-(L)1, we found that Tumor Volume_baseline_ predicts the outcome of anti-PD-(L)1 response (OS, p-value: 0.005, PFS, p-value:<0.001). We could thus consider to have much greater benefit of anti-PD-(L)1 treatment for patients in adjuvant setting with microscopic or no residual disease as compared to patients that had already received numerous lines of therapies (34).

Alternatively, the RECIST_baseline_ was not a prognostic biomarker. Therefore, the accurate measure of metastatic spread would be important to predict anti-PD-(L)1 response. Whether RECIST_baseline_ could be associated with ICB response still remains elusive given that some centers have also found that RECIST_baseline_ was not a prognostic biomarker (35), while others found the opposite (36, 37). Thus, more studies are needed to assess the role of RECIST_baseline_ as a predictive biomarker for ICB response. Furthermore, the RECIST choice of target lesions seems to suffer from reproducibility, as shown by Kuhl et al. (28) where 59% of the patients evaluated by three different radiologists had a discordance in the choice of the target lesions, resulting in different sums of diameters of five target lesions.

RECIST 1.1 criteria defines a progressive disease as an increase of 20% of the sum of the targeted lesions, a partial response as a decrease of 30% and a stable disease if none of the above (with the caveat of considering only the target lesions) (24). In this study, we chose to compare an increase or decrease of the RECIST diameters (RECIST_variation_) to an increase or decrease of the global volume (Volume_variation_), instead of a comparison to the RECIST 1.1 thresholds. Three key reasons justified this choice. First, and foremost, the median time of response or progression according to the RECIST 1.1 criteria was 3 months, while the time of the first evaluation was 2 months. Thus, using RECIST 1.1 criteria during the first evaluation was likely too restrictive as several patients would be considered with a stable disease while they could respond later. Second, while RECIST criteria had defined cut-offs to apply in the 1D setting, there is no consensus to define a response or a progression according to a 3D setting yet. Schiavon G. et al. examine this issue by mathematically transposing the RECIST thresholds to 3D (38). The concern of this approach is that the RECIST 1.1 only considers 5 lesions, whilst the total volume considers all the tumors in the body, which would imply that such a method would likely underestimate the total volume progression of all tumors. The Quantitative Imaging Biomarkers Alliance (QIBA) claims that a true change in lung tumor volume occurs at a specific threshold given a certain size. This study suggests that site-specific analysis should be performed to assess the repeatability and change detectability of tumor volume measurements (39). Third, since one of the key aim of this study was to compare the ability of RECIST and Volume to predict PFS/OS, we wanted to find a common threshold for both with a biological relevance. Both RECIST_variation_ and Volume_variation_ were predictive factors for PFS and OS, which is an argument in favor of this comparison.

Interestingly, Tumor Volume_variation_ did not show higher performances as compared to RECIST_variation_ suggesting that the extensive work of measuring all the lesions might be only necessary at baseline. However, we believe that Tumor Volume_variation_ assessment could be critical during treatment. Since the PFS is defined by the RECIST 1.1 criteria, the comparison between Tumor Volume_variation_ and RECIST_variation_ should only be done according to the OS. While Tumor Volume_variation_ accounts for non-target and new lesions, RECIST_variation_ does not. Interestingly, these lesions had been described to be at least as important as the target lesions determined by RECIST 1.1 in the occurrence of progressive disease (40, 41). Therefore, Volume_variation_ likely gives a more robust criteria for defining progression.

As previously reported in Nebot-Bral et al., NLR_baseline_ is highly variable across MSI tumors and should not be used as a predictive biomarker of ICB response (16). In this cohort, we confirmed NLR_variation_ as an early predictive biomarker. NLR_variation_, Volume_variation_ and RECIST_variation_ were not correlated. Consequently, these parameters should be used together to predict more accurately the outcome of anti-PD-(L)1 response shortly after treatment initiation. The concordance score is an easy, cost-effective way to combine clinical and biologic parameters for each patient.

The results of the phase II CheckMate-142 trial indicated that Nivolumab (anti-PD-1 monoclonal antibodies) plus Ipilimumab (anti-CTLA-4 monoclonal antibodies) achieved higher response rates than previously reported for nivolumab (55% vs. 31% ORR) in patients with pretreated MSI-H/MMRD metastatic CRC (42). Given that Volume_baseline_ and concordance score could be used as a prognostic and predictive biomarker of anti-PD-(L)1 therapy respectively for patients with MSI tumors, we could propose the combination of anti-PD-(L)1 + anti CTLA-4 either for patients with Volume _baseline_ > 90cm^3^ or patients that have an early increase of the concordance score. These results warrant further investigations on larger prospective cohorts.

Specific sites of the disease may hold unique mechanisms of resistance. Liver metastases have been associated with worse prognosis as opposed to lung metastases that are correlated with improved overall survival (43, 44). However, we did not find any correlation between the anatomical locations of metastases and ICB response. This could be due to the high heterogeneity of the cohort, with more than 10 different metastatic sites for 61 patients included in the study. In line with this, the hypothesis of resistance mechanisms being organ specific or texture parameters of the lesions discriminating response *vs* relapse, needs to be explored. Therefore, the use of radiomics to extract radiographic characteristics from the tumor image to produce statistics related to the heterogeneity or contrast of the tumor region, coupled with the predictive power of artificial intelligence (AI) algorithms is proposed as a new novel non-invasive biomarker for immunotherapy response (41–43).

This study has inherent limitations, including those that arise due to its retrospective nature and the performance being a single center study. Multi-center prospective cohort studies with larger sample sizes are warranted in the future. In addition, the NLR can be influenced by multiple factors, such as infection or drug uptake, which were not assessed in this study. Lastly, the volume of each tumor was approximated. Altogether, our results suggest that, for patients with MSI tumors, the use of tumor volume at baseline (Volume_baseline_) and the concomitant evaluation of NLR_variation_ and RECIST_variation_ or NLR_variation_ and Volume_variation_ earlier after treatment initiation predicts the outcome of anti-PD-(L)1 even before classical RECIST 1.1 evaluation of response/resistance.

## Data availability statement

The original contributions presented in the study are included in the article/**Supplementary Material**. Further inquiries can be directed to the corresponding authors.

## Ethics statement

The studies involving human participants were reviewed and approved by Gustave Roussy’s ethics committee. The patients/participants provided their written informed consent to participate in this study.

## Author contributions

Conceptualization: AH, NL, LN-B, and YB. Methodology: LN-B, YB, LL. Investigation: AH and LN-B. Resources: MK, LL, and LN-B. Funding acquisition: AH and NL. Project administration: AH, NL, HT, and P-HC. Writing – original draft: LN-B, YB, and LL. Writing – review & editing: YB, LN-B, LL, MK, CD, SA, P-HC, HT, PK, NC, NL, and AH. All authors contributed to the article and approved the submitted version.

## Funding

This work was funded by Gustave Roussy Cancer Campus, Centre National de la Recherche Scientifique (CNRS), SIRIC SOCRATE INCa-DGOS-INSERM_6043, SIRIC SOCRATE 2.0 INCa-DGOS-INSERM_12551, and La Ligue Nationale Contre le Cancer – Equipe labellisée EL2018_Kannouche. LN-B received funding from Philanthropia Foundation - Lombard Odier and la Ligue Nationale Contre le Cancer.

## Acknowledgments

This work was supported by DATAIA institute, Université Paris-Saclay, which provided the funding for the employment of YB and LL.

## Conflict of interest

NC reports grants from Cytune Pharma, grants from BMS, grants from SANOFI, personal fees from AstraZeneca France, outside the submitted work.

The remaining authors declare that the research was conducted in the absence of any commercial or financial relationships that could be construed as a potential conflict of interest.

## Publisher’s note

All claims expressed in this article are solely those of the authors and do not necessarily represent those of their affiliated organizations, or those of the publisher, the editors and the reviewers. Any product that may be evaluated in this article, or claim that may be made by its manufacturer, is not guaranteed or endorsed by the publisher.

## References

[1] Chen S Zhang Z Zheng X Tao H Zhang S Ma J. Response efficacy of PD-1 and PD-L1 inhibitors in clinical trials: A systematic review and meta-analysis. Front Oncol (2021) 11. doi: 10.3389/fonc.2021.562315 PMC8085334 33937012

[2] Sun L Zhang L Yu J Zhang Y Pang X Ma C. Clinical efficacy and safety of anti-PD-1/PD-L1 inhibitors for the treatment of advanced or metastatic cancer: a systematic review and meta-analysis. Sci Rep (2020) 10(1):2083. doi: 10.1038/s41598-020-58674-4 32034198 PMC7005709

[3] Zhao B Zhao H Zhao J. Efficacy of PD-1/PD-L1 blockade monotherapy in clinical trials. Ther Adv Med Oncol (2020) 12:1758835920937612. doi: 10.1177/1758835920937612 32728392 PMC7366397

[4] Debele TA Yeh CF Su WP. Cancer immunotherapy and application of nanoparticles in cancers immunotherapy as the delivery of immunotherapeutic agents and as the immunomodulators. Cancers (2020) 12(12):3773. doi: 10.3390/cancers12123773 PMC7765190 33333816

[5] Overman MJ McDermott R Leach JL Lonardi S Lenz HJ Morse MA. Nivolumab in patients with metastatic DNA mismatch repair-deficient or microsatellite instability-high colorectal cancer (CheckMate 142): an open-label, multicentre, phase 2 study. Lancet Oncol (2017) 18(9):1182–91. doi: 10.1016/S1470-2045(17)30422-9 PMC6207072 28734759

[6] Kim JH Kim SY Baek JY Cha YJ Ahn JB Kim HS. A phase II study of avelumab monotherapy in patients with mismatch repair-deficient/microsatellite instability-high or POLE-mutated metastatic or unresectable colorectal cancer. Cancer Res Treat (2020) 52(4):1135–44. doi: 10.4143/crt.2020.218 PMC7577804 32340084

[7] Le DT Durham JN Smith KN Wang H Bartlett BR Aulakh LK. Mismatch-repair deficiency predicts response of solid tumors to PD-1 blockade. Science (2017) 357(6349):409–13, eaan6733. doi: 10.1126/science.aan6733 PMC5576142 28596308

[8] Chalmers ZR Connelly CF Fabrizio D Gay L Ali SM Ennis R. Analysis of 100,000 human cancer genomes reveals the landscape of tumor mutational burden. Genome Med (2017) 9:34. doi: 10.1186/s13073-017-0424-2 28420421 PMC5395719

[9] The Cancer Genome Atlas Network. Comprehensive molecular characterization of human colon and rectal cancer. Nature (2012) 487(7407):330–7. doi: 10.1038/nature11252 PMC3401966 22810696

[10] Nebot-Bral L Brandao D Verlingue L Rouleau E Caron O Despras E. Hypermutated tumours in the era of immunotherapy: The paradigm of personalised medicine. Eur J Cancer (2017) 84(Supplement C):290–303. doi: 10.1016/j.ejca.2017.07.026 28846956

[11] Antill Y Kok PS Robledo K Yip S Cummins M Smith D. Clinical activity of durvalumab for patients with advanced mismatch repair-deficient and repair-proficient endometrial cancer. a nonrandomized phase 2 clinical trial. J Immunother Cancer (2021) 9(6). doi: 10.1136/jitc-2020-002255 PMC8190057 34103352

[12] Konstantinopoulos PA Luo W Liu JF Gulhan DC Krasner C Ishizuka JJ. Phase II study of avelumab in patients with mismatch repair deficient and mismatch repair proficient Recurrent/Persistent endometrial cancer. J Clin Oncol Off J Am Soc Clin Oncol (2019) 37(30):2786–94. doi: 10.1200/JCO.19.01021 PMC9798913 31461377

[13] André T Shiu KK Kim TW Jensen BV Jensen LH Punt C. Pembrolizumab in Microsatellite-Instability–high advanced colorectal cancer. N Engl J Med (2020) 383(23):2207–18. doi: 10.1056/NEJMoa2017699 33264544

[14] Azad NS Gray RJ Overman MJ Schoenfeld JD Mitchell EP Zwiebel JA. Nivolumab is effective in mismatch repair-deficient noncolorectal cancers: Results from arm Z1D-a subprotocol of the NCI-MATCH (EAY131) study. J Clin Oncol Off J Am Soc Clin Oncol (2019) 38(3):214–22. doi: 10.1200/jco.19.00818 PMC6968795 31765263

[15] Marabelle A Le DT Ascierto PA Di Giacomo AM De Jesus-Acosta A Delord JP. Efficacy of pembrolizumab in patients with noncolorectal high microsatellite Instability/Mismatch repair–deficient cancer: Results from the phase II KEYNOTE-158 study. J Clin Oncol (2019) 38(1):1–10. doi: 10.1200/jco.19.02105 PMC8184060 31682550

[16] Nebot-Bral L Hollebecque A Yurchenko AA de Forceville L Danjou M Jouniaux JM. Overcoming resistance to αPD-1 of MMR-deficient tumors with high tumor-induced neutrophils levels by combination of αCTLA-4 and αPD-1 blockers. J Immunother Cancer (2022) 10(7):e005059. doi: 10.1136/jitc-2022-005059 35896284 PMC9335020

[17] Bai R Lv Z Xu D Cui J. Predictive biomarkers for cancer immunotherapy with immune checkpoint inhibitors. biomark Res (2020) 8(1):34. doi: 10.1186/s40364-020-00209-0 32864131 PMC7450548

[18] Havel JJ Chowell D Chan TA. The evolving landscape of biomarkers for checkpoint inhibitor immunotherapy. Nat Rev Cancer. (2019) 19(3):133–50. doi: 10.1038/s41568-019-0116-x PMC6705396 30755690

[19] Charoentong P Finotello F Angelova M Mayer C Efremova M Rieder D. Pan-cancer immunogenomic analyses reveal genotype-immunophenotype relationships and predictors of response to checkpoint blockade. Cell Rep (2017) 18(1):248–62. doi: 10.1016/j.celrep.2016.12.019 28052254

[20] Chen DS Mellman I. Elements of cancer immunity and the cancer-immune set point. Nature. (2017) 541(7637):321–30. doi: 10.1038/nature21349 28102259

[21] Schalper KA Carleton M Zhou M Chen T Feng Y Huang SP. Elevated serum interleukin-8 is associated with enhanced intratumor neutrophils and reduced clinical benefit of immune-checkpoint inhibitors. Nat Med (2020) 26(5):688–92. doi: 10.1038/s41591-020-0856-x PMC8127102 32405062

[22] Gopalakrishnan V Spencer CN Nezi L Reuben A Andrews MC Karpinets TV. Gut microbiome modulates response to anti-PD-1 immunotherapy in melanoma patients. Science. (2018) 359(6371):97–103. doi: 10.1126/science.aan4236 29097493 PMC5827966

[23] Dall’Olio FG Marabelle A Caramella C Garcia C Aldea M Chaput N. Tumour burden and efficacy of immune-checkpoint inhibitors. Nat Rev Clin Oncol (2021), 1–16. doi: 10.1038/s41571-021-00564-3 34642484

[24] Eisenhauer EA Therasse P Bogaerts J Schwartz LH Sargent D Ford R. New response evaluation criteria in solid tumours: Revised RECIST guideline (version 1.1). Eur J Cancer. (2009) 45(2):228–47. doi: 10.1016/j.ejca.2008.10.026 19097774

[25] Champiat S Dercle L Ammari S Massard C Hollebecque A Postel-Vinay S. Hyperprogressive disease is a new pattern of progression in cancer patients treated by anti-PD-1/PD-L1. Clin Cancer Res Off J Am Assoc Cancer Res (2017) 23(8):1920–8. doi: 10.1158/1078-0432.CCR-16-1741 27827313

[26] Ferrara R Caramella C Besse B Champiat S. Pseudoprogression in non-small cell lung cancer upon immunotherapy: Few drops in the ocean? J Thorac Oncol Off Publ Int Assoc Study Lung Cancer (2019) 14(3):328–31. doi: 10.1016/j.jtho.2018.12.011 30782378

[27] Seymour L Bogaerts J Perrone A Ford R Schwartz LH Mandrekar S. iRECIST: guidelines for response criteria for use in trials testing immunotherapeutics. Lancet Oncol (2017) 18(3):e143–52. doi: 10.1016/S1470-2045(17)30074-8 PMC5648544 28271869

[28] Kuhl CK Alparslan Y Schmoee J Sequeira B Keulers A Brümmendorf TH. Validity of RECIST version 1.1 for response assessment in metastatic cancer: A prospective, multireader study. Radiology. (2019) 290(2):349–56. doi: 10.1148/radiol.2018180648 30398433

[29] van Griethuysen JJM Fedorov A Parmar C Hosny A Aucoin N Narayan V. Computational radiomics system to decode the radiographic phenotype. Cancer Res (2017) 77(21):e104–7. doi: 10.1158/0008-5472.CAN-17-0339 PMC5672828 29092951

[30] Capone M Giannarelli D Mallardo D Madonna G Festino L Grimaldi AM. Baseline neutrophil-to-lymphocyte ratio (NLR) and derived NLR could predict overall survival in patients with advanced melanoma treated with nivolumab. J Immunother Cancer. (2018) 6(1):74. doi: 10.1186/s40425-018-0383-1 30012216 PMC6048712

[31] Ferrucci PF Gandini S Battaglia A Alfieri S Di Giacomo AM Giannarelli D. Baseline neutrophil-to-lymphocyte ratio is associated with outcome of ipilimumab-treated metastatic melanoma patients. Br J Cancer. (2015) 112(12):1904–10. doi: 10.1038/bjc.2015.180 PMC4580390 26010413

[32] Davidson-Pilon C. Lifelines, survival analysis in Python. Zenodo (2022) 4(40):1317. Available at: https://zenodo.org/record/6359609. doi: 10.21105/joss.01317

[33] Reback J McKinney W jbrockmendel Bossche JVd Augspurger T Cloud P. Pandas-dev/pandas: Pandas 1.0.3. Zenodo (2020). Available at: https://zenodo.org/record/3715232.

[34] ESMO. Moving immunotherapy to the adjuvant setting . Available at: https://www.esmo.org/meetings/past-meetings/esmo-immuno-oncology-congress-2017/educational-articles/Moving-Immunotherapy-to-the-Adjuvant-Setting.

[35] Lenci E Marcantognini G Cognigni V Lupi A Rinaldi S Cantini L. Tumor burden as possible biomarker of outcome in advanced NSCLC patients treated with immunotherapy: a single center, retrospective, real-world analysis. Explor Target Anti-Tumor Ther (2021) 2(3):227–39. doi: 10.37349/etat.2021.00043 PMC9400786 36046436

[36] Joseph RW Elassaiss-Schaap J Kefford R Hwu WJ Wolchok JD Joshua AM. Baseline tumor size is an independent prognostic factor for overall survival in patients with melanoma treated with pembrolizumab. Clin Cancer Res (2018) 24(20):4960–7. doi: 10.1158/1078-0432.CCR-17-2386 PMC6916264 29685882

[37] Katsurada M Nagano T Tachihara M Kiriu T Furukawa K Koyama K. Baseline tumor size as a predictive and prognostic factor of immune checkpoint inhibitor therapy for non-small cell lung cancer. Anticancer Res (2019) 39(2):815–25. doi: 10.21873/anticanres.13180 30711962

[38] Schiavon G Ruggiero A Schöffski P van der Holt B Bekers DJ Eechoute K. Tumor volume as an alternative response measurement for imatinib treated GIST patients. PloS One (2012) 7(11):e48372. doi: 10.1371/journal.pone.0048372 23133631 PMC3487791

[39] QIBACTVol_TumorVolumeChange(2022)-20220721.pdf . Available at: https://qibawiki.rsna.org/images/b/ba/QIBACTVol_TumorVolumeChange%282022%29-20220721.pdf.

[40] Litière S de Vries EGE Seymour L Sargent D Shankar L Bogaerts J. The components of progression as explanatory variables for overall survival in the response evaluation criteria in solid tumours 1.1 database. Eur J Cancer Oxf Engl 1990. (2014) 50(10):1847–53. doi: 10.1016/j.ejca.2014.03.014 24726734

[41] Fournier L de Geus-Oei LF Regge D Oprea-Lager DE D’Anastasi M Bidaut L. Twenty years on: RECIST as a biomarker of response in solid tumours an EORTC imaging group - ESOI joint paper. Front Oncol (2021) 11:800547. doi: 10.3389/fonc.2021.800547 35083155 PMC8784734

[42] Overman MJ Lonardi S Wong KYM Lenz HJ Gelsomino F Aglietta M. Durable clinical benefit with nivolumab plus ipilimumab in DNA mismatch repair-Deficient/Microsatellite instability-high metastatic colorectal cancer. J Clin Oncol Off J Am Soc Clin Oncol (2018) 36(8):773–9. doi: 10.1200/JCO.2017.76.9901 29355075

[43] Pires da Silva I Lo S Quek C Gonzalez M Carlino MS Long GV. Site-specific response patterns, pseudoprogression, and acquired resistance in patients with melanoma treated with ipilimumab combined with anti–PD-1 therapy. Cancer. (2020) 126(1):86–97. doi: 10.1002/cncr.32522 31584722

[44] Yu J Green MD Li S Sun Y Journey SN Choi JE. Liver metastasis restrains immunotherapy efficacy *via* macrophage-mediated T cell elimination. Nat Med (2021) 27:152–64. doi: 10.1038/s41591-020-1131-x PMC8095049 33398162

