# Supplementary material for: Predicting immunotherapy outcomes in patients with MSI tumors using NLR and CT global tumor volume
Source: Front Oncol. 2022 Oct 25;12:982790. doi: 10.3389/fonc.2022.982790 (PMC9641225; doi:10.3389/fonc.2022.982790)
Supplement: Supplementary file 1 [file DataSheet_1.docx]

Supplementary Material


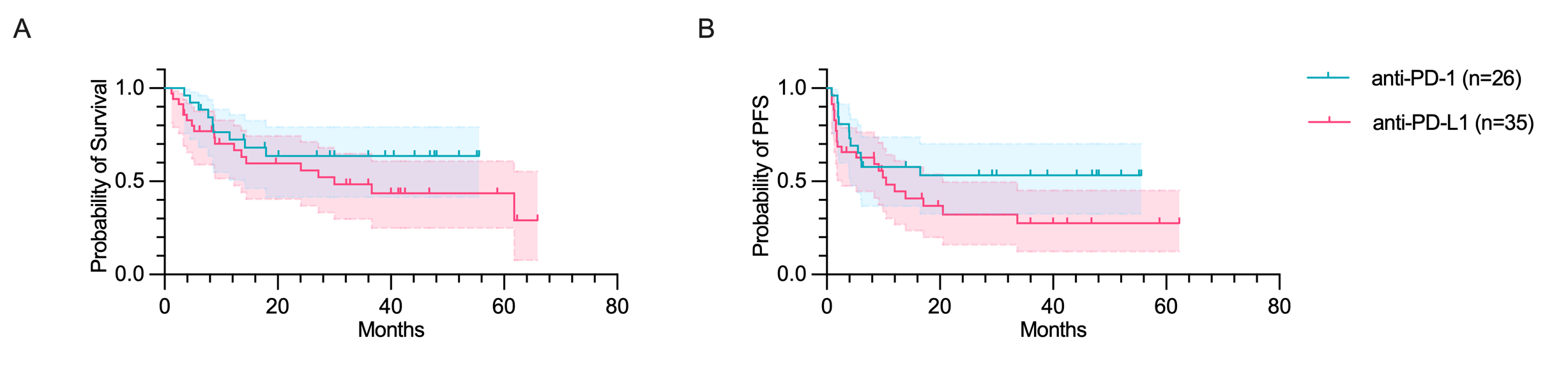


**Supplementary Figure 1.** No significant difference in OS or PFS according to anti-PD-1 or anti-PDL-1 treatment used. Overall survival (A) and Progression-free survival (B) plotted using a Kaplan Meier estimation with their respective confidence intervals (95%). For Kaplan–Meier estimation, tick marks represent data censored at the time of the last imaging assessment and statistical analyses were performed using Log-rank (Mantel-Cox) test. Symbol significance: * p≤0.05; ** p≤0.01; *** ≤0.001, **** ≤0.0001.


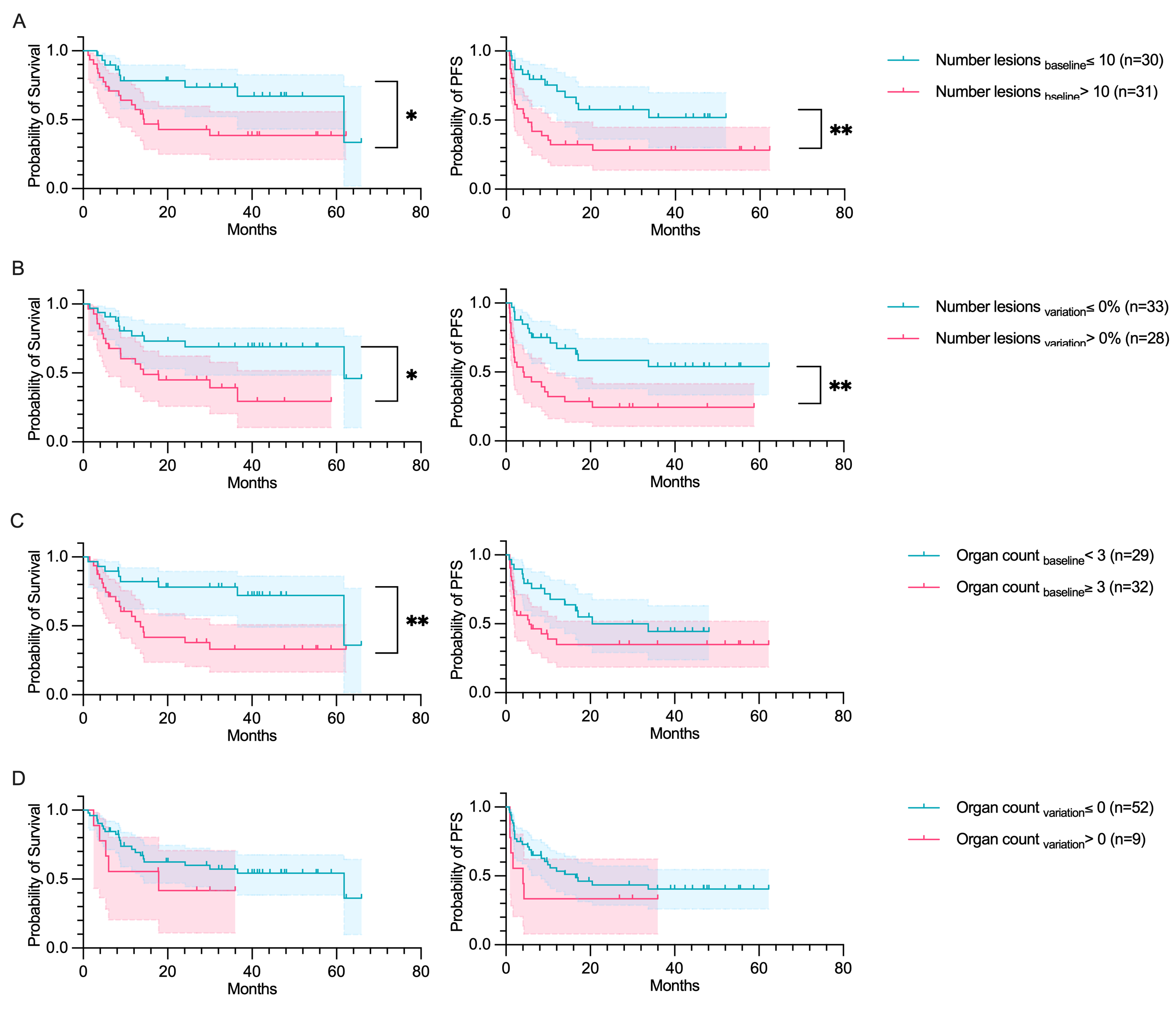


**Supplementary Figure 2.** Overall survival and Progression-free survival plotted using a Kaplan Meier estimation according to A) Number lesions _baseline_, B) Number lesions _variation_, C) Organ count _baseline_, D) Organ count _variation_. For Kaplan–Meier estimation, tick marks represent data censored at the time of the last imaging assessment and statistical analyses were performed using Log-rank (Mantel-Cox) test. Symbol significance: * p≤0.05; ** p≤0.01; *** ≤0.001, **** ≤0.0001.
